# Muscle wasting in chronic kidney disease: the role of the ubiquitin proteasome system and its clinical impact

**DOI:** 10.1007/s00467-007-0594-z

**Published:** 2008-04-01

**Authors:** Vik R. Rajan, William E. Mitch

**Affiliations:** Nephrology Division M/S: BCM 285, Baylor College of Medicine, One Baylor, Plaza, Alkek N-520, Houston, TX 77030 USA

**Keywords:** Ubiquitin-proteasome system (UPS), Caspase-3, 14kD actin fragment, Muscle wasting, Protein degradation, Muscle atrophy, Uremia, Chronic kidney disease (CKD)

## Abstract

Muscle wasting in chronic kidney disease (CKD) and other catabolic diseases (e.g. sepsis, diabetes, cancer) can occur despite adequate nutritional intake. It is now known that complications of these various disorders, including acidosis, insulin resistance, inflammation, and increased glucocorticoid and angiotensin II production, all activate the ubiquitin–proteasome system (UPS) to degrade muscle proteins. The initial step in this process is activation of caspase-3 to cleave the myofibril into its components (actin, myosin, troponin, and tropomyosin). Caspase-3 is required because the UPS minimally degrades the myofibril but rapidly degrades its component proteins. Caspase-3 activity is easily detected because it leaves a characteristic 14kD actin fragment in muscle samples. Preliminary evidence from several experimental models of catabolic diseases, as well as from studies in patients, indicates that this fragment could be a useful biomarker because it correlates well with the degree of muscle degradation in dialysis patients and in other catabolic conditions.

## Maintenance of protein stores in chronic kidney disease

In uremic patients, protein stores are frequently depressed when assessed by a low serum prealbumin and weight loss, which includes loss of muscle mass [1, 2]. In pediatric patients with chronic kidney disease (CKD), linear growth is impaired and muscle mass is reduced. Although these findings have been attributed to “malnutrition”, many uremic patients with muscle wasting have not developed the problems because of inadequate diet; instead, they have complications that induce a complex series of physiological and biochemical adaptations, resulting in protein catabolism [3, 4]. In children and adults with CKD, these complications include metabolic acidosis, insulin resistance, increased glucocorticoid production, high levels of angiotensin II (Ang II), and inflammation [5–8]. Many observational studies and mechanistic investigations have attempted to explain this loss of protein stores, and especially the loss of muscle mass. There are at least three conclusions from these studies. First, rates of protein synthesis in muscle are generally unchanged, whereas rates of protein degradation tend to increase [5, 9]. Second, the daily rates of protein turnover in cells are so high (3.5–4.5 g protein/kg per day) that even a small increase in protein degradation (and/or a decrease in protein synthesis) will cause marked protein depletion over time [10]. Third, the increase in muscle protein degradation in uremia and most other catabolic disease states is mostly due to programmed activation of the ubiquitin–proteasome system (UPS) [5, 11]. Therefore, to understand muscle wasting, one must understand the UPS.

## The ATP-dependent, ubiquitin–proteasome system (UPS)

**Fig. 1:**
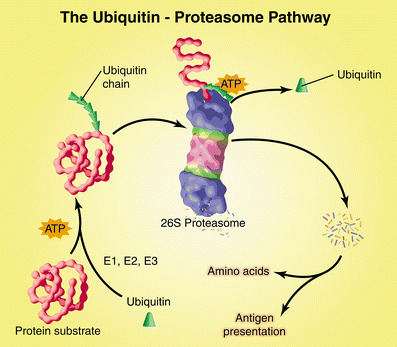
The ubiquitin–proteasome pathway of protein degradation. Ubiquitin (Ub) is conjugated to proteins destined for degradation by an ATP-dependent process that involves three enzymes (E1–E3). A chain of five Ub molecules attached to the protein substrate is recognized by the 26S proteasome, which removes Ub and digests the protein into peptides. The peptides are degraded to amino acids by peptidases in the cytoplasm or used in antigen presentation. (Reproduced with permission from [15])

Over the past two decades, progress in understanding the action and regulation of the UPS has been at the center of attempts to understand the control of protein turnover. The UPS includes concerted actions of enzymes that link ubiquitin (Ub), a member of the heat-shock protein family, to protein substrates that are destined for degradation (Fig. 1). When a chain of at least four to five ubiquitins are linked to a protein, it is marked for degradation in a second step mediated by the proteasome [12, 13]. Specifically, the tagged protein will be recognized by the 26S proteasome, a very large multicatalytic protease complex that not only recognizes Ub-conjugated proteins but also removes Ub, unwinds the protein, and injects it into the central core of the 26S proteasome. Once inside this central, tube-like structure, the protein substrate is degraded into small peptides [14].

Three enzymatic components are required to link Ub to proteins that are destined for degradation. There appears to be only one E1 (Ub-activating) enzyme and around 40 E2 (Ub-carrier or conjugating) proteins. The key enzyme, however, is the E3 enzyme, which accounts for the exquisite specificity of proteins to be degraded. There are at least a thousand E3 enzymes (Ub ligase), and each can recognize a specific protein substrate and catalyze the transfer of an activated Ub from the E2 carrier protein to the substrate protein [10, 15].

Since the initial reports that the UPS recognizes specific proteins and tags them for destruction, knowledge about proteolytic processes in the proteasome has exploded. Thousands of proteins have been recognized as being degraded by the UPS, and novel cellular functions are now known to be regulated by Ub conjugation. In terms of protein breakdown, the major functions of the pathway are:

### Rapid removal of proteins

Protein degradation is irreversible, and hence, destruction of a protein generally leads to a complete termination of cellular process mediated by the protein. Consequently, protein degradation is critical for the regulation of metabolism and cell turnover. The rapid degradation of specific proteins also permits cells (as well as the organism) to rapidly adapt to a change in physiological conditions (e.g. requiring a switch to glucose as an energy source involves converting protein stores into amino acids that can be used in gluconeogenesis).

### Regulation of gene transcription

Ub conjugation affects gene transcription because many transcription factors become conjugated to Ub, and transcription activators are degraded by the proteasome [16]. This process regulates transcriptional activity by removing “spent” activators and resetting a promoter for additional rounds of transcription [17]. Second, the ability of transcription factors to function varies with their location within the cell. For example, nuclear factor (NF)-κB, a proinflammatory transcriptional factor, is kept outside the nucleus because movement into the nucleus is blocked by its association with an inhibitory protein, IκB. Destruction of IκB, initiated by the IKK kinase and carried out by the UPS, frees NF-κB, which then translocates to the nucleus to stimulate gene transcription [18]. A clinical application of this function of the UPS has developed in oncology. Bortezomib (Velcade, PS-341), a proteasome inhibitor, has proven to be beneficial in patients with multiple myeloma and is currently in clinical trials for the treatment of other cancers [19, 20]. The proposed mechanism of action involves the ability of bortezomib to prevent the UPS-induced destruction of IκB, thereby blocking the activation of NF-κB (an antiapoptosis transcription factor), leading to an increase in apoptosis [21]. Inhibition of the proteasome, therefore, will induce apoptosis of the neoplastic cells [22]. In addition, myeloma cells are also particularly dependent upon NF-κB to produce essential growth factors [especially inerleukin 6 (Il-6)]; when NF-κB is inactive, the growth of myeloma cells is depressed.

### Quality-control mechanism

The UPS selectively eliminates abnormally folded or damaged proteins that have arisen because of missense or nonsense mutations, biosynthetic errors, proteins damaged by oxygen radicals, or by denaturation. For example, in cystic fibrosis, the mutant form of the transmembrane conductance regulator protein (CFTR) is selectively degraded before it reaches the cell surface [23]. The UPS catalyzes destruction of this mutant CFTR because its tertiary structure is abnormal. Another example is the degradation of misfolded proteins within the endoplasmic reticulum. Endoplasmic-reticulum-associated degradation (ERAD) of proteins removes misfolded proteins by targeting them for destruction by proteasomes in the cytoplasm [24].

### Influencing the function of the immune system

The UPS is responsible for creating antigens from the degradation of foreign proteins (e.g. viral particles). The antigens are presented on the major histocompatibility complex as class I molecules. In this way, the 26S proteasome exerts dual roles of removing foreign proteins and creating a stimulus of the immune system [15, 25].

### As a source of amino acids

When carbohydrate calories are rapidly needed or when cells must respond to catabolic diseases/conditions, there is breakdown of cell proteins, especially skeletal muscle proteins. The UPS degrades muscle proteins to provide amino acids that can be converted to glucose (i.e. gluconeogenesis). An undesired consequence of this activity could be an inappropriate loss of muscle protein.

### Functions of Ub not associated with proteolysis

Ub can also be conjugated to proteins as a monomer (rather than as the typical Ub chain). When this occurs on cell-surface proteins, the protein is internalized into the endocytic pathway to be degraded in lysosomes [26, 27].

## Uremia-activated mechanisms that accelerate loss of muscle protein

Results from rodent models of CKD have established that accelerated muscle protein catabolism involves many of the same cellular mechanisms that cause muscle wasting in other catabolic conditions, such as cancer, starvation, insulin deficiency/resistance, or sepsis [10, 28]. The principal mechanism causing muscle atrophy in CKD involves activation of the UPS. Evidence for this includes the presence of higher levels of mRNAs encoding certain components of the UPS, as well as a similar pattern of increases and decreases in the expression of about 100 atrophy-related genes (also called atrogenes) [5, 28]. Changes in atrogenes include decreased expression of various growth-related genes and increased expression of components of the UPS. Patients with different clinical conditions associated with muscle atrophy exhibit similar increases in mRNAs encoding components of the UPS (e.g. an increase in mRNAs encoding Ub and proteasome subunits) [11, 29–31]. In these cases, changes in gene expression are most likely due to transcriptional regulation because we have shown that uremia or abnormal insulin responses increase the transcription of Ub and subunits of the proteasome [5, 6, 32]. Additional evidence linking the UPS to protein degradation in catabolism is the finding that the increase in protein degradation in the muscle of rats with CKD (and other muscle-wasting conditions) can be blocked by inhibitors of the proteasome [5, 6, 33]. Considered together, these results indicate that muscle wasting is a specific and carefully orchestrated program.

Other questions are why are proteins degraded, and how is the complex program triggered in widely varied pathological conditions (e.g. acidosis in renal failure, low insulin levels in fasting and diabetes, inactivity, or glucocorticoids and cytokines in sepsis and other inflammatory conditions)? In fasting and in other disease states, acceleration of muscle-protein breakdown mobilizes amino acids, which are used for protein synthesis in tissues and for conversion to glucose in the liver [10]. However, if excessive protein degradation persists, the protein loss will have deleterious effects. In CKD, the breakdown of tissue protein produces nitrogenous waste products, which must be excreted to prevent the accumulation of uremic toxins [3, 10]. Finally, in muscle wasting conditions, contractile proteins are lost differentially, whereas in conditions causing atrophy (e.g. aging), all components of muscle cells seem to be affected.

What accounts for muscle-specific response? The answer to this question lies in the involvement of the UPS. The UPS degrades a specific protein depending on which E3 ubiquitin ligase is activated. For example, two Ub ligases, atrogin-1 (also known as MAFbx) and MuRF-1, are found specifically in muscle, and their expression increases dramatically (8- to 20-fold) in catabolic states, causing loss of muscle protein [28, 34, 35]. In mice lacking the genes for either atrogin-1 or MuRF-1, muscles grow normally, but in response to muscle denervation, the ensuing atrophy is 30–50% slower [34]. In addition, the muscle’s content of atrogin-1 mRNA can be considered a biomarker for the rate of proteolysis in muscles responding to a catabolic condition [36–38].

## In uremia, the initial cleavage of myofibrillar proteins is mediated by caspase-3

Myofibrillar proteins (including actomyosin) comprise about two thirds of the protein in muscle, the major store of amino acids for new protein synthesis and for gluconeogenesis. The UPS readily degrades the main proteins in myofibrils (i.e. actin, myosin, troponin, or tropomyosin), but it does not readily break up the myofibril into its main component proteins [39]. This means that another proteolytic system must initially digest myofibrils to create substrates that can be degraded by the UPS [40].

Many catabolic conditions are associated with inflammation and/or cell injury, and these conditions activate a cysteine protease called caspase-3. We tested caspase-3 in an in vitro system using purified actomyosin and found that caspase-3 cleaves actomyosin and leaves a characteristic 14kD actin fragment [40]. When we activated caspase-3 in cultured muscle cells, we found that UPS rapidly degraded myofibrillar component proteins, and again, the 14kD C-terminal fragment of actin was left in the muscle cells [40]. The protein-cleaving action of caspase-3 is important because blocking caspase-3 will reduce overall protein degradation in muscle [40]. In addition, we have found the same cleavage processes are present in muscles of rodent models of uremia, type-1 or type-2 diabetes, or in Ang-II-induced hypertension [7, 35, 40, 41]. Moreover, we found that the 14kD actin fragment also accumulates in muscles of patients with loss of muscle mass due to painful osteodystrophy, uremia, or burn injury [42]. In the latter study, we found that the rate of protein degradation in muscle (measured from the turnover of labeled amino acids) directly correlated (*r* = 0.78) with the level of the 14kD actin fragment in the same muscle. In addition, there was a lower level of the 14kD actin fragment in muscle of hemodialysis patients who participated in 18 weeks of an endurance exercise training program. In summary, the level of the 14kD actin fragment is directly associated with measured protein degradation in muscle, and the accumulation of the fragment responds to a beneficial therapeutic intervention. If these properties hold up in other trials, the level of the 14kD actin fragment could be used as a “biomarker” of increased muscle protein degradation [42].

## Signals triggering muscle atrophy in kidney disease and other catabolic states

Complications of CKD, as well as the complex syndrome of uremia, can trigger muscle protein breakdown. The triggering complications include metabolic acidosis, decreased insulin action (including insulin resistance), increased glucocorticoid production, high levels of Ang II, and inflammation [5–8]. Metabolic acidosis is known to cause accelerated protein degradation in infants, children, adults, the elderly, and patients with CKD (Table 1). The mechanism by which metabolic acidosis causes muscle wasting involves activation of the UPS and caspase-3 [40, 43]. In addition, acidosis changes hormone actions, such as insulin resistance and increased glucocorticoid production, which are involved in activating protein degradation [32, 41, 43–45] (Table 2). It is important to emphasize that the correction of metabolic acidosis has been shown to decrease protein breakdown in various clinical trials, indicating why maintaining normal serum bicarbonate levels should be part of standard clinical care (Table 1).

**Table 1:** Evidence that metabolic acidosis induces catabolism of protein and amino acids in normal infants, children, and adults, as well as in patients with chronic kidney disease (CKD)

Subjects investigated	Outcome measurements	Trial outcome
Infants [68]	Low-birth-weight, acidotic infants were given NaHCO_3_ or NaCl	NaHCO_3_ supplement improved growth
Children with CKD [69]	Measured rates of protein degradation in children with CKD	Protein loss was ∼ 2-fold higher when HCO_3_ was < 16 mM compared with > 22.6 mM
Normal adults [70]	Acidosis induced and then measured amino acid and protein metabolism	Acidosis increased amino acid and protein degradation
Normal adults [71]	Induced acidosis and then measured nitrogen balance and albumin synthesis	Acidosis induced negative nitrogen balance and suppressed albumin synthesis
Chronic renal failure [72]	Nitrogen balance before and after treatment of acidosis	NaHCO_3_ improved nitrogen balance
Chronic renal failure [73]	Essential amino acid and protein degradation before and after treatment of acidosis	NaHCO_3_ suppressed amino acid and protein degradation
Chronic renal failure [74]	Muscle protein degradation and degree of acidosis	Proteolysis was proportional to acidosis and blood cortisol
Chronic renal failure [75]	Nitrogen balance before and after treatment of acidosis	NaHCO_3_ reduced urea production and improved nitrogen balance
Hemodialysis [76]	Protein degradation before and after treatment of acidosis	NaHCO_3_ decreased protein degradation
Hemodialysis [77]	Serum albumin before and after treatment of acidosis	NaHCO_3_ increased serum albumin
CAPD [78]	Protein degradation before and after treatment of acidosis	NaHCO_3_ decreased protein degradation
CAPD [79]	Weight and muscle gain before and after treatment of acidosis	Raising dialysis buffer increased weight and muscle mass

*CAPD* continuous ambulatory peritoneal dialysis

**Table 2:** Metabolic acidosis in otherwise normal humans changed hormonal levels or responses to hormones

Hormone	Acidosis-induced response
Growth hormone (GH) [80–84]	Suppressed GH secretion
	Lower IGF-1 response
Insulin [44, 85, 86]	Suppressed insulin-stimulated glucose metabolism
Insulin-like growth factor (IGF)-1 [81, 84, 87]	Decreased IGF-1 in plasma, and kidney and liver (but not in muscle)
Thyroid hormone [82, 88]	Decreased plasma T_3_ and T_4_ levels plus a higher plasma thyroid-stimulating hormone
Glucocorticoids [89]	Increased glucocorticoid production
Parathyroid hormone (PTH) [90, 91]	Decreased sensitivity of PTH secretion to changes in plasma calcium
Vitamin D [91]	Suppressed activation to 1,25 (OH)_2_ cholecalciferol

**Fig. 2:**
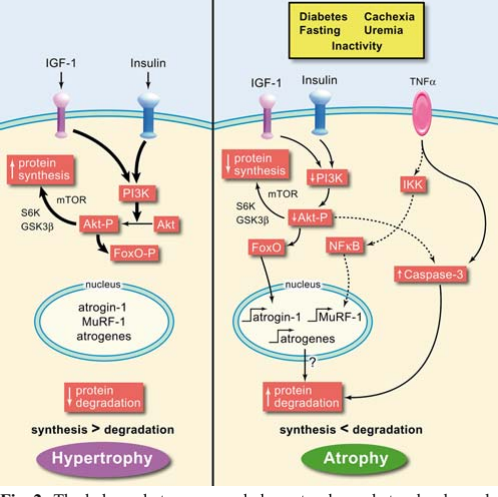
The balance between muscle hypertrophy and atrophy depends on whether protein synthesis is more active than degradation or vice versa. In protein synthesis, insulin-like growth factor (IGF)-1 and insulin signaling leads to increased phosphatidylinositol 3-kinase (PI3K), which promotes Akt phosphorylation (Akt-P). Akt-P promotes phosphorylation of GSK1 and mTOR/S6 kinases, leading to increased protein synthesis. Akt-P also phosphorylates the forkhead (FoxO) transcription factor, preventing it from entering the nucleus to promote expression of atrogin-1, MuRF-1, and other atrogenes, thereby blocking protein degradation. In protein degradation, the opposite pathway happens, but additionally, decreased Akt-P leads to increased caspase-3 activity, further promoting degradation. In inflammation, it is thought that tumor necrosis factor (TNF)-α and other inflammatory cytokines phosphorylate the inhibitor of nuclear factor (NF)-κB (IκB), to free NF-B to enter the nucleus and promote MuRF-1 expression, and ultimately, muscle protein degradation. (Reproduced with permission from [15])

The finding that various diseases with muscle wasting are caused by activation of the UPS, plus the fact that coordinated changes in the expression of genes in muscle occur in different catabolic states, suggest that catabolic states activate a common cellular signaling pathway [28]. One signaling pathway is a decrease in phosphatidylinositol 3-kinase (PI3K) activity (Fig. 2). The involvement of this signaling pathway follows from the finding that several catabolic illnesses, including sepsis, acidosis, uremia, and diabetes, are characterized by insulin resistance [41, 44, 46, 47]. In normal muscle, binding of insulin or insulin-like growth factor (IGF)-1 to their receptors increases the activities of PI3K and its downstream target, Akt. In insulin-resistant conditions or with deficiency of IGF-1, the activity of this signaling pathway is depressed [35, 48, 49]. When PI3K activity falls, there is decreased production of phosphatidylinositol-3,4,5 phosphate (PIP3), leading to decreased phosphorylation and activity of the downstream serine/threonine kinase, Akt. This is a key step, because activated Akt is a major stimulator of growth-related processes via phosphorylation of the downstream kinases, GSK1 and mTOR/S6kinase, stimulating protein synthesis. On the other hand, reduced PI3K-Akt signaling (as occurs in insulin resistance) enhances protein breakdown in muscle [41]. The rise in muscle protein losses is associated with two catalytic processes: first, caspase-3 is activated to break down the complex structure of muscle; second, there is increased expression of the E3 ubiquitin ligases, atrogin-1 and MuRF-1, to degrade the proteins made available by caspase-3 [35–37] (Fig. 2). The result is muscle wasting.

How are the two mechanisms activated? In studies of muscles from insulin-deficient rats or db/db mice (a model of insulin resistance), we found that accelerated muscle protein degradation increases the level of the proapoptotic factor, Bax [35, 41]. This is relevant because activated Bax causes the release of cytochrome *C* from mitochondria. Cytochrome *C* release, in turn, activates caspase-3 to trigger actomyosin/myofibril cleavage, leaving behind the 14kD actin fragment marker [35]. The activation of atrogin-1, and hence the UPS, involves another mechanism: changes in activity of the fork-head transcription factors (FoxO 1, 3 and 4). When these transcription factors are phosphorylated by Akt, they cannot enter the nucleus to stimulate transcription of atrogin-1. However, when PI3K/Akt activities are low, the FoxOs are not phosphorylated, so they can enter the nucleus to increase the transcription of atrogin-1, resulting in an increase in muscle protein degradation [35–37].

The influence of Akt on the other E3 ubiquitin ligase involved in muscle proteolysis, MuRF-1, is not as clearly established, and it may be linked to inflammation, as activation of NF-κB will cause overproduction of MuRF1 and muscle atrophy [37].

Besides acidosis and depressed insulin/IGF-1 action, another complication of CKD that participates in muscle wasting is increased glucocorticoid production. The complexity of these interactions is great because increased glucocorticoids can cause insulin insensitivity [32, 41, 50], and both insulin deficiency and insulin resistance increase glucocorticoid production. Glucocorticoids exert a permissive effect on protein degradation in muscle caused by several catabolic conditions. For example, activation of muscle protein breakdown does not occur in adrenalectomized animals with metabolic acidosis or with acute diabetes unless the animals are also given a physiological dose of glucocorticoids [32, 51–53]. Similarly, the increase in muscle proteolysis induced by Ang II or sepsis is largely eliminated by inhibiting the glucocorticoid receptor [7, 54]. This response to glucocorticoids is “permissive” because the same physiological level of glucocorticoids does not stimulate muscle protein degradation unless the animals are either acidotic or insulin deficient. These complex interactions actually make “physiological sense” because glucocorticoids evolved to integrate stress responses in different tissues. When glucose is needed, glucocorticoids mobilize amino acids from muscle protein. At the same time, glucocorticoids induce gluconeogenic enzymes in liver to catalyze the conversion of the amino acids to glucose and urea.

In children, accelerated loss of protein stores associated with glucocorticoid therapy frequently results in impaired linear growth. One glucocorticoid-dependent mechanism causing these defects is impaired response to insulin (insulin insensitivity). An allied mechanism is impaired response to IGF-1 arising from decreased or impaired action of growth hormone. Indeed, administration of growth hormone has been shown to improve linear growth of children being treated chronically with glucocorticoids [55]. Moreover, growth hormone has been shown to improve growth of children with chronic catabolic diseases such as CKD; whereas growth hormone improves growth, it does not improve growth in children to (or close to) a normal height for age [56–58]. The impaired response is likely due to the multiple complications of CKD, resulting in retarded growth. Interestingly, there is a small uncontrolled study of five children who were being treated with chronic intermittent hemodialysis and growth hormone. The children were then placed on an intensified daily hemodialysis regimen (3 h/day, five to six times a week) for a median time of 1.5 years [59]. The new regimen led to significant catchup growth into the range of a normal height for age. Intensified dialysis plus growth hormone could correct acidosis, improve insulin and IGF-1 signaling and responses to other hormones, and remove unidentified uremic toxins. These responses emphasize the complexity of sorting out mechanisms for muscle wasting in CKD.

Another catabolic factor associated with CKD is Ang II. Infusion of Ang II into rodents causes both anorexia and muscle protein loss by mechanisms that depend on glucocorticoids [7]. Also, there is the knotty problem of understanding the influence of inflammation. In this case, the mechanism(s) causing muscle wasting in inflammation is not clear. Suggested responses include the ability of certain inflammatory mediators to cause insulin resistance, as well as a more direct influence of inflammatory mediators on muscle protein metabolism (Fig. 2). The problem is difficult because the link between an increase in inflammatory markers in uremic patients (e.g. C-reactive protein) to loss of muscle mass has not been established [60, 61].

Finally, there is an intriguing protein—myostatin—a member of the transforming growth factor (TGF)-β family of cytokines. It is produced in skeletal and cardiac muscle and regulates muscle growth by limiting it [62]. Overexpression of myostatin in muscle leads to loss of protein mass via inhibition of Akt phosphorylation with an increase in active FoxO1; this increases the expression of atrophy-related genes [63, 64]. Underexpression of myostatin results in skeletal muscle hypertrophy [65]. An increase in myostatin expression is found in several cachexia-associated disease states. However, there is limited information about the influence of kidney disease on myostatin expression and function beyond changes in myostatin mRNA [66, 67].

## Conclusion

In this brief review of mechanisms causing muscle protein losses, we discussed how a complex series of biochemical reactions are coordinated to create a genetic program that degrades muscle proteins. We also identified an initial step in muscle proteolysis that leaves behind a biomarker in muscle, the 14kD actin fragment, resulting from caspase-3. We emphasized how the UPS causes muscle wasting in uremia, as well as the role UPS plays in the regulation of cellular functions, ranging from the control of the cell cycle to activities that promote cancer. Indeed, inhibitors of proteasome activity have emerged as novel chemotherapeutic agents. Involvement of the UPS in such a wide range of functions explains why the 2004 Nobel Prize in Chemistry was awarded to Avram Hershko, Aaron Ciechanover, and Irwin Rose for their discovery of Ub and its role in orchestrating cellular protein turnover (https://doi.org/nobelprize.org/chemistry/laureates/2004/).

## References

[1] Qureshi AR, Alvestrand A, Danielsson A, Divino-Filho JC, Gutierrez A, Lindholm B, Bergstrom J (1998). Factors predicting malnutrition in hemodialysis patients: A cross-sectional study. Kidney Int.

[2] Kopple JD (2001). National Kidney Foundation K/DOQI clinical practice guidelines for nutrition in chronic renal failure. Am J Kidney Dis.

[3] Mitch WE (2002). Malnutrition: a frequent misdiagnosis for hemodialysis patients. J Clin Invest.

[4] Kaysen GA, Dubin JA, Muller H-G, Rosales L, Levin NW, Mitch WE (2004). Inflammation and reduced albumin synthesis associated with stable decline in serum albumin in hemodialysis patients. Kidney Int.

[5] Bailey JL, Wang X, England BK, Price SR, Ding X, Mitch WE (1996). The acidosis of chronic renal failure activates muscle proteolysis in rats by augmenting transcription of genes encoding proteins of the ATP-dependent, ubiquitin-proteasome pathway. J Clin Invest.

[6] Price SR, Bailey JL, Wang X, Jurkovitz C, England BK, Ding X, Phillips LS, Mitch WE (1996). Muscle wasting in insulinopenic rats results from activation of the ATP-dependent, ubiquitin-proteasome pathway by a mechanism including gene transcription. J Clin Invest.

[7] Song Y-H, Li Y, Du J, Mitch WE, Rosenthal N, Delafontaine P (2005). Muscle-specific expression of insulin-like growth factor-1 blocks angiotensin II-induced skeletal muscle wasting. J Clin Invest.

[8] Stenvinkel P, Heimburger O, Paultre F, Diczfalusy U, Wang T, Berglund L, Jogestrand T (1999). Strong association between malnutrition, inflammation and atherosclerosis in chronic kidney failure. Kidney Int.

[9] Lim VS, Kopple JD (2000). Protein metabolism in patients with chronic renal failure: Role of uremia and dialysis. Kidney Int.

[10] Mitch WE, Goldberg AL (1996). Mechanisms of muscle wasting: The role of the ubiquitin-proteasome system. N Engl J Med.

[11] Pickering WP, Price SR, Bircher G, Marinovic AC, Mitch WE, Walls J (2002). Nutrition in CAPD: Serum bicarbonate and the ubiquitin-proteasome system in muscle. Kidney Int.

[12] Glickman MH, Ciechanover A (2002). The ubiquitin-proteasome proteolytic pathway: destruction for the sake of construction. Physiol Rev.

[13] Pickart CM (2004). Back to the future with ubiquitin. Cell.

[14] Baumeister W, Walz J, Zuhl F, Seemuller E (1998). The proteasome: paradigm of a self-compartmentalizing protease. Cell.

[15] Lecker SH, Goldberg AL, Mitch WE (2006). Protein degradation by the ubiquitin-proteasome pathway in normal and disease states. J Am Soc Nephrol.

[16] Muratani M, Tansey WP (2003). How the ubiquitin-proteasome system controls transcription. Nat Rev Mol Cell Biol.

[17] Lipford JR, Smith GT, Chi Y, Deshaies RJ (2005). A putative stimulatory role for activator turnover in gene expression. Nature.

[18] Karin M, Ben-Neriah Y (2000). Phosphorylation meets ubiquitination: the control of NF-[kappa]B activity. Annu Rev Immunol.

[19] Kisselev AF, Akopian TN, Woo KM, Goldberg AL (1999). The sizes of peptides generated from protein by mammalian 26 and 20 S proteasomes. Implications for understanding the degradative mechanism and antigen presentation. J Biol Chem.

[20] Adams J (2004). The proteasome: a suitable antineoplastic target. Nat Rev Cancer.

[21] Palombella VJ, Rando OJ, Goldberg AL, Maniatis T (1994). The ubiquitin-proteasome pathway is required for processing the NF-kB1 precursor protein and the activation of NF-kB. Cell.

[22] Kisselev AF, Goldberg AL (2001). Proteasome inhibitors: from research tools to drug candidates. Chem Biol.

[23] Jensen TJ, Loo MA, Pind S, Williams DB, Goldberg AL, Riordan JR (1995). Multiple proteolytic systems, including the proteasome contribute CFTR processing. Cell.

[24] Meusser B, Hirsch C, Jarosch E, Sommer T (2005). ERAD: the long road to destruction. Nat Cell Biol.

[25] Rock KL, Gramm C, Rothstein L, Clark K, Stein R, Dick L, Hwang D, Goldberg AL (1994). Inhibitors of the proteasome block the degradation of most cell proteins and the generation of peptides presented on MHC class 1 molecules. Cell.

[26] Hicke L, Dunn R (2003). Regulation of membrane protein transport by ubiquitin and ubiquitin-binding proteins. Annu Rev Cell Dev Biol.

[27] Sigismund S, Polo S, Di Fiore PP (2004). Signaling through monoubiquitination. Curr Top Microbiol Immunol.

[28] Lecker SH, Jagoe RT, Gomes M, Baracos V, Bailey JL, Price SR, Mitch WE, Goldberg AL (2004). Multiple types of skeletal muscle atrophy involve a common program of changes in gene expression. FASEB J.

[29] Mansoor O, Beaufrere Y, Boirie Y, Ralliere C, Taillandier D, Aurousseau E, Schoeffler P, Arnal M, Attaix D (1996). Increased mRNA levels for components of the lysosomal, Ca++-activated and ATP-ubiquitin-dependent proteolytic pathways in skeletal muscle from head trauma patients. Proc Natl Acad Sci USA.

[30] Tiao G, Hobler S, Wang JJ, Meyer TA, Luchette FA, Fischer JE, Hasselgren P-O (1997). Sepsis is associated with increased mRNAs of the ubiquitin-proteasome proteolytic pathway in human skeletal muscle. J Clin Invest.

[31] Williams AB, Sun X, Fischer JE, Hasselgren P-O (1999). The expression of genes in the ubiquitin-proteasome proteolytic pathway is increased in skeletal muscle from patients with cancer. Surgery.

[32] Mitch WE, Bailey JL, Wang X, Jurkovitz C, Newby D, Price SR (1999). Evaluation of signals activating ubiquitin-proteasome proteolysis in a model of muscle wasting. Am J Physiol.

[33] Tawa NE, Odessey R, Goldberg AL (1997). Inhibitors of the proteasome reduce the accelerated proteolysis in atrophying rat skeletal muscles. J Clin Invest.

[34] Bodine SC, Latres E, Baumhueter S, Lai VK, Nunez L, Clark BA, Poueymirou WT, Panaro FJ, Na E, Dharmarajan K, Pan ZQ, Valenzuel DM, DeChiara TM, Stitt TN, Yancopoulos GD, Glass DJ (2001). Identification of ubiquitin ligases required for skeletal muscle atrophy. Science.

[35] Lee SW, Dai G, Hu Z, Wang X, Du J, Mitch WE (2004). Regulation of muscle protein degradation: coordinated control of apoptotic and ubiquitin-proteasome systems by phosphatidylinositol 3 kinase. J Am Soc Nephrol.

[36] Sandri M, Sandri C, Gilbert A, Skuck C, Calabria E, Picard A, Walsh K, Schiaffino S, Lecker SH, Goldberg AL (2004). Foxo transcription factors induce the atrophy-related ubiquitin ligase atrogin-1 and cause skeletal muscle atrophy. Cell.

[37] Stitt TN, Drujan D, Clarke BA, Panaro F, Timofeyva Y, Kline WO, Gonzalez M, Yancopoulos GD, Glass DJ (2004). The IGF-1/PI3K/Akt pathway prevents expression of muscle atrophy-induced ubiquitin ligases by inhibiting FOXO transcription factors. Mol Cell.

[38] Sacheck JM, Ohtsuka A, McLary SC, Goldberg AL (2004). IGF-1 stimulates muscle growth by suppressing protein breakdown and expression of atrophy-related ubiquitin ligases, atrogin-1 and MuRF1. Am J Physiol.

[39] Solomon V, Goldberg AL (1996). Importance of the ATP-ubiquitin-proteasome pathway in degradation of soluble and myofibrillar proteins in rabbit muscle extracts. J Biol Chem.

[40] Du J, Wang X, Meireles CL, Bailey JL, Debigare R, Zheng B, Price SR, Mitch WE (2004). Activation of caspase 3 is an initial step triggering muscle proteolysis in catabolic conditions. J Clin Invest.

[41] Wang XH, Hu Z, Hu JP, Du J, Mitch WE (2006). Insulin resistance accelerates muscle protein degradation: activation of the ubiquitin-proteasome pathway by defects in muscle cell signaling. Endocrinology.

[42] Workeneh B, Rondon-Berrios H, Zhang L, Hu Z, Ayehu G, Ferrando A, Kopple JD, Wang H, Storer TW, Fournier M, Lee SW, Du J, Mitch WE (2006). Development of a diagnostic method for detecting increased muscle protein degradation in patients with catabolic conditions. J Am Soc Nephrol.

[43] Mitch WE, Medina R, Greiber S, May RC, England BK, Price SR, Bailey JL, Goldberg AL (1994). Metabolic acidosis stimulates muscle protein degradation by activating the ATP-dependent pathway involving ubiquitin and proteasomes. J Clin Invest.

[44] DeFronzo RA, Beckles AD (1979). Glucose intolerance following chronic metabolic acidosis in man. Am J Physiol.

[45] May RC, Bailey JL, Mitch WE, Masud T, England BK (1996). Glucocorticoids and acidosis stimulate protein and amino acid catabolism in vivo. Kidney Int.

[46] Hasselgren P-O, Warner BW, James H, Takehara H, Fischer JE (1987). Effect of insulin on amino acid uptake and protein turnover in skeletal muscle from septic rats: Evidence for insulin resistance of protein degradation. Arch Surg.

[47] Siew ED, Pupim LB, Majchrzak KM, Shintani A, Flakoll PJ, Ikizler TA (2007). Insulin resistance is associated with skeletal muscle protein breakdown in non-diabetic chronic hemodialysis patients. Kidney Int.

[48] Bodine SC, Stitt TN, Gonzalez M, Kline WO, Stover GL, Bauerlein R, Zlotchenko E, Scrimgeour A, Lawrence JC, Glass DJ, Yancopoulos GD (2001). Akt/mTOR pathway is a crucial regulator of skeletal muscle hypertrophy and can prevent muscle atrophy in vivo. Nat Cell Biol.

[49] Bailey JL, Price SR, Zheng B, Hu Z, Mitch WE (2006). Chronic kidney disease causes defects in signaling through the insulin receptor substrate/phosphatidylinositol 3-kinase/Akt pathway: implications for muscle atrophy. J Am Soc Nephrol.

[50] Saad MJ, Folli F, Kahn JA, Kahn CR (1993). Modulation of insulin receptor, insulin receptor substrate-1, and phosphatidylinositol 3-kinase in liver and muscle of dexamethasone-treated rats. J Clin Invest.

[51] May RC, Kelly RA, Mitch WE (1986). Metabolic acidosis stimulates protein degradation in rat muscle by a glucocorticoid-dependent mechanism. J Clin Invest.

[52] Wing SS, Goldberg AL (1993). Glucocorticoids activate the ATP-ubiquitin-dependent proteolytic system in skeletal muscle during fasting. Am J Physiol.

[53] Price SR, England BK, Bailey JL, Van Vreede K, Mitch WE (1994). Acidosis and glucocorticoids concomitantly increase ubiquitin and proteasome subunit mRNAs in rat muscle. Am J Physiol.

[54] Tiao G, Fagan J, Roegner V, Lieberman M, Wang J-J, Fischer JE, Hasselgren P-O (1996). Energy-ubiquitin-dependent muscle proteolysis during sepsis in rats is regulated by glucocorticoids. J Clin Invest.

[55] Mauras N (2001). Growth hormone therapy in the glucocorticosteroid-dependent child: metabolic and linear growth effects. Horm Res.

[56] Neu AM, Bedinger M, Fivush BA, Warady BA, Watkins SL, Friedman AL, Brem AS, Goldstein SL, Frankenfield DL (2005). Growth in adolescent hemodialysis patients: data from the Centers for Medicare & Medicaid Services ESRD Clinical Performance Measures Project. Pediatr Nephrol.

[57] Mehls O, Schaefer F, Tonshoff B, Wuhl E (2002). Effectiveness of growth hormone treatment in short children with chronic renal failure. J Pediatr.

[58] Fine RN, Sullivan EK, Tejani A (2000). The impact of recombinant human growth hormone treatment on final adult height. Pediatr Nephrol.

[59] Fischbach M, Terzic J, Menouer S, Dheu C, Soskin S, Helmstetter A, Burger MC (2006). Intensified and daily hemodialysis in children might improve statural growth. Pediatr Nephrol.

[60] Cai D, Frantz JD, Tawa NE, Melendez PA, Oh BC, Lidov HG, Hasselgren PO, Frontera WR, Lee J, Glass DJ, Shoelson SE (2004). IKKbeta/NF-kappaB activation causes severe muscle wasting in mice. Cell.

[61] Cai D, Yuan M, Frantz DF, Melendez PA, Hansen L, Lee J, Shoelson SE (2005). Local and systemic insulin resistance resulting from hepatic activation of IKK-beta and NF-kappaB. Nat Med.

[62] Lee SJ (2004). Regulation of muscle mass by myostatin. Annu Rev Cell Dev Biol.

[63] Zimmers TA, Davies MV, Koniaris LG, Haynes P, Esquela AF, Tomkinson KN, McPherron AC, Wolfman NM, Lee S-J (2002). Induction of cachexia in mice by systemically administered myostatin. Science.

[64] McFarlane C, Plummer E, Thomas M, Hennebry A, Ashby M, Ling N, Smith H, Sharma M, Kambadur R (2006). Myostatin induces cachexia by activating the ubiquitin proteolytic system through an NF-kappaB-independent, FoxO1-dependent mechanism. J Cell Physiol.

[65] Schuelke M, Wagner KR, Stolz LE, Hubner C, Riebel T, Komen W, Braun T, Tobin JF, Lee SJ (2004). Myostatin mutation associated with gross muscle hypertrophy in a child. N Engl J Med.

[66] Sun DF, Xheng Z, Tummala P, Oh J, Schaefer F, Rabkin R (2004). Chronic uremia attenuates growth hormone-induced signal transduction in skeletal muscle. J Am Soc Nephrol.

[67] Wang H, Casaburi R, Taylor WE, Aboellail H, Storer TW, Kopple JD (2005). Skeletal muscle mRNA for IGF-IEa, IGF-II, and IGF-I receptor is decreased in sedentary chronic hemodialysis patients. Kidney Int.

[68] Kalhoff H, Diekmann L, Kunz C, Stock GJ, Manz F (1997). Alkali therapy versus sodium chloride supplement in low birthweight infants with incipient late metabolic acidosis. Acta Paediatr.

[69] Boirie Y., Broyer M., Gagnadoux M.F., Niaudet P., Bresson J-L (2000). Alterations of protein metabolism by metabolic acidosis in children with chronic renal failure. Kidney Int.

[70] Reaich D, Channon SM, Scrimgeour CM, Goodship TH (1992). Ammonium chloride-induced acidosis increases protein breakdown and amino acid oxidation in humans. Am J Physiol.

[71] Ballmer PE, McNurlan MA, Hulter HN, Anderson SE, Garlick PJ, Krapf R (1995). Chronic metabolic acidosis decreases albumin synthesis and induces negative nitrogen balance in humans. J Clin Invest.

[72] Papadoyannakis NJ, Stefanidis CJ, McGeown M (1984). The effect of the correction of metabolic acidosis on nitrogen and protein balance of patients with chronic renal failure. Am J Clin Nutr.

[73] Reaich D, Channon SM, Scrimgeour CM, Daley SE, Wilkinson R, Goodship TH (1993). Correction of acidosis in humans with CRF decreases protein degradation and amino acid oxidation. Am J Physiol.

[74] Garibotto G, Russo R, Sofia A, Sala MR, Robaudo C, Moscatelli P, DeFerrari G, Tizianello A (1994). Skeletal muscle protein synthesis and degradation in patients with chronic renal failure. Kidney Int.

[75] Williams B, Hattersley J, Layward E, Walls J (1991). Metabolic acidosis and skeletal muscle adaptation to low protein diets in chronic uremia. Kidney Int.

[76] Graham KA, Reaich D, Channon SM, Downie S, Goodship TH (1997). Correction of acidosis in hemodialysis decreases whole-body protein degradation. J Am Soc Nephrol.

[77] Movilli E, Zani R, Carli O, Sangalli L, Pola A, Camerini C, Cancarini GC, Scolari F, Feller P, Maiorca R (1998). Correction of metabolic acidosis increases serum albumin concentration and decreases kinetically evaluated protein intake in hemodialysis patients: A prospective study. Nephrol Dial Transplant.

[78] Graham KA, Reaich D, Channon SM, Downie S, Gilmour E, Passlick-Deetjen J, Goodship TH (1996). Correction of acidosis in CAPD decreases whole body protein degradation. Kidney Int.

[79] Stein A, Moorhouse J, Iles-Smith H, Baker R, Johnstone J, James G, Troughton J, Bircher G, Walls J (1997). Role of an improvement in acid-base status and nutrition in CAPD patients. Kidney Int.

[80] Challa A, Krieg RJ, Thabet MA, Veldhuis JD, Chan JC (1993). Metabolic acidosis inhibits growth hormone secretion in rats: mechanism of growth retardation. Am J Physiol.

[81] Brungger M, Hulter HN, Krapf R (1997). Effect of chronic metabolic acidosis on the growth hormone/IGF-1 endocrine axis: New cause of growth hormone insensitivity in humans. Kidney Int.

[82] Wiederkehr MR, Kalogiros J, Krapf R (2004). Correction of metabolic acidosis improves thyroid and growth hormone axes in haemodialysis patients. Nephrol Dial Transplant.

[83] Kuemmerle N, Krieg RJ, Latta K, Challa A, Hanna JD, Chan JC (1997). Growth hormone and insulin-like growth factor in non-uremic acidosis and uremic acidosis. Kidney Int.

[84] Green J, Maor G (2002). Effect of metabolic acidosis on the growth hormone/IGF-1 endocrine axis in skeletal growth centers. Kidney Int.

[85] Kobayashi S, Maesato K, Moriya H, Ohtake T, Ikeda T (2005). Insulin resistance in patients with chronic kidney disease. Am J Kidney Dis.

[86] Mak RH (1998). Effect of metabolic acidosis on insulin action and secretion in uremia. Kidney Int.

[87] Bereket A, Wilson TA, Kolasa AJ, Fan J, Lang CH (1996). Regulation of the insulin-like growth factor system by acute acidosis. Endocrinology.

[88] Brungger M, Hulter HN, Krapf R (1997). Effect of chronic metabolic acidosis on thyroid hormone homeostasis in humans. Am J Physiol.

[89] Schambelan M, Sebastian A, Katuna A, Arteaga E (2001). Adrenocortical hormone secretory response to chronic NH4Cl-induced metabolic acidosis. Am J Physiol.

[90] Graham KA, Reaich D, Channon SM, Downie S, Goodship TH (1997). Correction of acidosis in hemodialysis patients increases the sensitivity of the parathyroid glands to calcium. J Am Soc Nephrol.

[91] Krapf R., Vetsch R., Vetsch W., Hulter HN (1992). Chronic metabolic acidosis increases the serum concentration of 1,25-dihydroxyvitamin D in humans by stimulating its production rate. J Clin Invest.

